# Viewpoint and practical recommendations from a movement disorder specialist panel on objective measurement in the clinical management of Parkinson’s disease

**DOI:** 10.1038/s41531-018-0051-7

**Published:** 2018-05-10

**Authors:** Per Odin, K. Ray Chaudhuri, Jens Volkmann, Angelo Antonini, Alexander Storch, Espen Dietrichs, Zvezdan Pirtošek, Tove Henriksen, Malcolm Horne, David Devos, Filip Bergquist

**Affiliations:** 1 University of Lund, Lund, Sweden; 2 University Hospital Reinkenheide, Bremerhaven, Germany; 3 National Parkinson Foundation (NPF) International Centre of Excellence, Department of Neurology, King’s College Hospital, London, UK; 4 Department of Basic and Clinical Neuroscience, The Maurice Wohl Clinical Neuroscience Institute, King’s College London, London, UK; 5 Department of Neurology, University Clinic of Würzburg, Würzburg, Germany; 6 Parkinson and Movement Disorders Unit, Department of Neuroscience, University of Padua, Padua, Italy; 7 Department of Neurology, University of Rostock, Rostock, Germany; 8 Department of Neurology, Oslo University Hospital and University of Oslo, Oslo, Norway; 9 Department of Neurology, University Medical Center, Ljubljana, Slovenia; 10 University Hospital of Bispebjerg, Copenhagen, Denmark; 11 Centre for Clinical Neurosciences and Neurological Research, St Vincent’s Hospital, Melbourne, Australia; 12 Florey Institute for Neuroscience and Mental Health, University of Melbourne, Parkville, VIC Australia; 13 Department of Medical Pharmacology, Lille University, INSERM UMRS_1171, University Hospital Center, LICEND COEN Center, Lille, France; 14 Department of Pharmacology, Institute of Neuroscience and Physiology, Sahlgrenska Academy, University of Gothenburg, Gothenburg, Sweden

## Abstract

Motor aspects of Parkinson’s disease, such as fluctuations and dyskinesia, can be reliably evaluated using a variety of “wearable” technologies, but practical guidance on objective measurement (OM) and the optimum use of these devices is lacking. Therefore, as a first step, a panel of movement disorder specialists met to provide guidance on how OM could be assessed and incorporated into clinical guidelines. A key aspect of the incorporation of OM into the management of Parkinson’s disease (PD) is defining cutoff values that separate “controlled” from “uncontrolled” symptoms that can be modified by therapy and that relate to an outcome that is relevant to the person with PD (such as quality of life). Defining cutoffs by consensus, which can be subsequently tested and refined, is the first step to optimizing OM in the management of PD. OM should be used by all clinicians that treat people with PD but the least experienced may find the most value, but this requires guidance from experts to allow non-experts to apply guidelines. While evidence is gained for devices that produce OM, expert opinion is needed to supplement the evidence base.

## Introduction

Motor and non-motor fluctuations in Parkinson’s disease (PD) are difficult to evaluate accurately,^1^ and while scales such as the wearing-off scale^2^ and Hauser diary provide an estimate, they suffer from being retrospective, subjective and affected by recall bias. Some motor aspects of PD can now be reliably evaluated using a variety of “wearable” technologies.^3–5^ This process of monitoring PD features with repeated measurements, particularly motor symptoms, is referred to as objective measurement (OM).

While there are many publications regarding OM in PD (recently reviewed in refs. ^3,4,6^), their focus has been principally on the *how* of measurement rather than its *use*, especially in routine clinical care. OM can be used as an end point of clinical trials or for diagnosis but its use in therapeutic management of PD is the focus here. Altman^7^ noted that measurement in clinical practice is so routine that it is taken for granted and its importance forgotten. Yet, OM has never been implemented in the routine clinical management of PD, and this could be perceived as an unmet need.

The potential impact of OM in PD can be gleaned by considering the principles of OM in managing other medical disorders, where its role is well established, and as discussed by Maetzler et al. (see Box 1).^8^

A panel of 11 internationally recognized movement disorder specialists (SP) in the care of PD met to consider the way OM could be used to improve clinical management in PD. This SP met four times between June 2014 and October 2016. The proposed principles outlined in Box 1 (based upon those discussed by Maetzler et al.)^8^ were discussed by the SP at the first meeting, with focus on their relevance to PD. It was agreed that four proposed principles in Box 1 would be relevant to PD and each was serially addressed over the course of the meetings. Each member of the SP was assigned a topic, on which they gathered information and presented at the meeting. Due to time-constraints, not all topics (for example, balance and gait) were discussed in detail at these meetings. There were also discussions and recommendations around practicalities of the use of OM in PD (section “Practical guidance on objective measurement in routine clinical care of PD?” below). The process was designed to achieve broad agreement and to be presented in the format below. A core team of authors developed the initial draft of this manuscript to reflect these discussions, and each author then reviewed the drafts for content until consensus was achieved. It is anticipated that this manuscript will be the first in a series of discussions on optimizing the clinical utility of new OM technology and is intended as both a guide and a starting point for future debate.

### Box 1. Proposed principles for OM that are relevant to PD^8–11^

1) OM is most valuable when there is a therapy that modifies the objectively measured disease indicator or a change in therapy can alleviate complications.

2) OM is only relevant when changes in the measured indicator relate to a change in outcomes.

3) There is a particular value of an objectively measured indicator that separates “controlled” from “uncontrolled” symptoms so that this value constitutes a therapeutic target.

4) OM may be superior to qualitative measures in determining whether a patient is controlled or uncontrolled.

## What should be measured in PD?

The first proposed principle in Box 1 is that OM is most valuable when there is a therapy that relates to the objectively measured disease indicator. As previously reported, PD symptoms and features that might be measured to reflect therapy effects include akinesia, dyskinesia, fluctuations, gait/instability, and physical inactivity.^8^ These can be grouped as treatment responsive symptoms and treatment complications.

Motor symptoms are the most responsive and current main target for PD therapy.^12^ Bradykinesia may currently be the most readily measured aspect of dopamine deficiency, and it can well act as a surrogate for those symptoms that are more difficult to measure. Tremor may also be a potential target for OM.

Of the adverse symptoms that result from, or that are exacerbated by, substituting dopamine deficiency in the brain, dyskinesia is an obvious target for OM. While OM may be possible for sleep,^13^ measuring dyskinesia seems the most imminent. It is likely that therapeutic interventions that reduce or delay the motor complications of therapy (i.e., fluctuations and/or dyskinesia) will also treat non-motor complications, and so, for the time being dyskinesia might act as a proxy for those treatment complications that develop from excessive dopaminergic treatment.

In addition to symptoms that respond to, or develop from, treatment there is an important group of PD symptoms that may become refractory to treatment as PD progresses. Dementia, classic freezing of gait, festination, postural instability, dysphagia, dysarthria, and gastro-paresis are examples; gait may initially respond well to treatment but be quite impervious to treatment at a later time. OM of gait may have a future role in the assessment of PD as there is evidence that some interventions may improve gait stability and balance.^14,15^ OM of gait parameters can already, in some specific situations, influence PD management decisions.^15,16^ If effective treatment is developed for any of these PD symptoms, they may become useful targets for OM.

### Panel consensus

Objectively measuring bradykinesia, tremor, and dyskinesia addresses proposed principle 1 in Box 1. Changes in bradykinesia, tremor, and dyskinesia reflect changes in dopamine-related brain activity patterns brought about by therapy.

## Does treatment of “treatment responsive symptoms” improve outcomes?

Changes in the measured indicator should relate to changes in clinically relevant outcomes, which are among others the individual’s health-related quality of life (HRQoL) or disease-related costs.^8^ There is evidence that more severe motor symptoms in PD are associated with worse HRQoL and higher costs, and improving motor symptoms with therapy improves HRQoL and reduces costs.^17–38^

### Panel consensus

The worse the bradykinesia, tremor, fluctuation, or dyskinesia, the lower the HRQoL and the greater the costs. Therapies that treat bradykinesia, tremor, fluctuation, and dyskinesia also improve HRQoL and reduce costs.

## Objective measurement leads to identification of therapeutic targets and therapeutic guidelines?

The third principle in Box 1 is that an OM is of particular value if it separates “controlled” from “uncontrolled” symptoms and this value constitutes a therapeutic target (i.e., intervention should aim to move the patient toward the controlled state or “normal range”).^8^

In PD, the therapeutic target is often insufficiently defined and may vary over the course of the disease. Treatment effects are assessed with rating scales, such as UPDRS, but there are no established levels in PD rating scales that are used to define therapeutic targets (other than a rarely achieved complete absence or “zero level”).

In other areas of medicine, OMs are used to establish whether a patient’s measured indicator lies within “target” because being within target has a better outcome. In conditions involving these indicators, therapeutic guidelines provide advice on how to shift the measured indicator from being in the “uncontrolled” range to the target range. However, it must be emphasized that measurements, targets, and guidelines do not replace clinical judgment in the management of the individual. If for example, there were objective measures for bradykinesia in PD, there may be several reasons in the individual case why bradykinesia scores lie outside a “target” range. People with Parkinson’s disease (PwP) may:

- respond poorly to dopaminergic therapy, suggestive of an atypical phenotype,
- respond well to dopaminergic therapy but treatment-related adverse events require withdrawal or contraindicate an increase in medications,
- present suboptimally treated symptoms, i.e., bradykinesia that was unrecognized by them, the carer or clinicians and therefore not perceived as something that could be improved, or because of the PwP’s wish to avoid side effects or complications.

It may seem obvious, but treatment can only be optimized if it is known that treatment is suboptimal and whether any intervention to address this suboptimal state produced the intended effects.

How a target score for a particular indicator can be established was discussed by Maetzler et al.^8^ Identification of target scores frequently begins as a consensus statement from experts, which in turn stimulates studies to test their views. It should be noted that even where evidence is advanced, such as in managing blood pressure, expert opinion is expressed in guidelines.^39^ A natural starting point for OM-guided therapy targets in PD will be the normal range in a healthy age-matched population.

Any potential OM of PD symptoms should have a reasonable correlation to the established and validated qualitative ordinal rating scales, like UPDRS and AIMS. Though OM can also evaluate symptoms that are not measurable with clinical scales. It is also necessary to demonstrate that potential OM are responsive to relevant treatment alterations. Furthermore, it is an advantage if the behavior of the OM in a healthy population is known. Once these conditions are established it is possible to define a crude treatment target, e.g., that the OM should be within the range observed in the healthy population mean ±1.96 SD or the healthy population interquartile range. In PD, we can predict however that very ambitious treatment targets may increase the risk of short- and long-term adverse effects, so the expert opinion on what is an appropriate treatment target with, for example, a bradykinesia OM may have to be tempered to a measure within the “worse half” of the normal range.

We identified currently available measurement systems via general web-based searches conducted using the terms “movement disorder monitoring,” “Parkinson’s disease, monitoring,” and “Parkinson’s disease, motion detection devices.” Searches were made for any such devices with FDA, EMA, and/or CE certification as medical devices. Two measurement systems are currently recognized by regulatory bodies and have CE marking to provide some assurance regarding safety, efficacy, and privacy.^40,41^ The Kinesia^TM^ system (manufactured by Great Lakes NeuroTechnologies, Valley View, OH) mainly focusing on tremor^3,42,43^ and the Parkinson’s KinetiGraph^TM^ (PKG^TM^; Global Kinetic Corporation, Melbourne, Australia) (Table 1).^44^ These certifications differentiate these medical devices from the many sensors that are available for scientific and personal use—such devices are discussed elsewhere.^3^

**Table 1:** Overview of the two FDA-approved OM devices

		Continuous assessment			
Device	Description	Bradykinesia	Dyskinesia	Tremor	Task-based
PKG^TM^	Wrist worn sensor	✓	✓	✓	✗
Kinesia™	Wrist and ankle sensor bands	✗	✓	✓	✓

In a pilot study, the Kinesia™ system demonstrated that it could be used successfully to monitor PD motor fluctuations, and could aid in evaluating the efficacy of treatment of the PwP.^45^ Furthermore, the measurements from the Kinesia™ system had a high correlation with clinicians’ scores for rest tremor (*r*(2) = 0.89), postural tremor (*r*(2) = 0.90), and kinetic tremor (*r*(2) = 0.69).^43^ These data suggest that it may be possible to define treatment targets for tremor using the Kinesia™ system.

PKG^TM^ bradykinesia OM and dyskinesia OM are correlated to UPDRS and AIMS, respectively, and are responsive to treatment changes.^46^ Furthermore, the normal ranges of these measures in healthy individuals have been reasonably well defined. Motor state measurements with PKG^TM^ also correlate on a daily basis with patient diaries—however, hour-to-hour correlation was weak.^47^ Interestingly, the authors suggested that disagreement between diaries and the OM may partially be caused by the fact that PKG^TM^ scores are continuous, whereas diaries have a three-point scale (“off,” “on,” or dyskinetic).^47^ Patients essentially select their own threshold to define these states, but such a threshold will vary from day to day. If we can define a specific threshold on a continuous OM, this can potentially improve assessment of these fluctuations. Therefore, a reasonable starting point to consider for an OM target would be the established “normal ranges.” However, as previously discussed, aiming for such a target in PD would risk over-treatment and adverse events, and therefore, we need to modify these targets using expert opinion. Based on his extensive practical experience with PKG^TM^, and on his co-authorship of the recent correlation study,^46^ M.H. was invited by the SP to propose such modified thresholds for the PKG^TM^. These targets were initially reviewed and modified by another member of the SP with comprehensive practical experience of PKG^TM^ (Filip Bergquist) before being discussed, amended, and ratified by the SP in the course of the meetings. The proposed PKG^TM^ bradykinesia OM and dyskinesia OM targets can also be compared with corresponding AIMS scores and UPDRS scores, based on the correlations observed by Griffiths et al.^46^ to verify that these are within an achievable range (see Glossary to Table 2). The proposed treatment targets are presented here as a first step to encourage future testing and refining (Table 2). A caveat to any such OM target is that a median score may not reflect the aspect of, for example, bradykinesia that is most important to the PwP. Clinical judgment and interpretation of OM will, therefore, remain central to clinical decisions, and future refinement of OM targets will help to establish what features are the most important.

**Table 2:** Possible targets for treating PD based on PKG^TM^ normal reference ranges^a^

**Bradykinesia**	
Optimally controlled	BKS <23
Acceptable control	BKS ≥23 and ≤25 and no fluctuations (see below)
Uncontrolled	BKS >25 (including marked PTI if BKS very high)
**Dyskinesia**	
Optimally controlled	DKS <7 and FDS <10.8
Acceptable control	DKS 7–9 and FDS <13 and no fluctuations (see below)
Uncontrolled	Median DKS >9
**Tremor**	
Optimally controlled	No detectable tremor—<1% of the day with oscillatory activity >10 s
Acceptable control	To be determined—insufficient data
Uncontrolled	Detectable tremor that disturbs the patient
**Immobility/sleep**	
Daytime sleep	To be determined—insufficient data
Nocturnal sleep	To be determined—insufficient data
**Impulse control behaviors**	
ICB risk	RR >200

Glossary and reference to aid interpretation of tables above			
**BKS refers to bradykinesia score on PKG** ^**TM**^		**DKS refers to dyskinesia score on PKG** ^**TM**^	
**BKS**	**~UPDRS III**	**DKS**	**~mAIMS**
21	14	5	5
23	21	10	10
25	27	15	15

BKS and DKS in the glossary above refer to the median values for the 6 days of recording

FDS = PKG^TM^ Fluctuation Dyskinesia Score: Interquartile range values for normal subject 7.8–12.8

PTI = Percent time immobility. In daytime, a PTI >5% indicates increased daytime sleepiness

RR = PKG^TM^ risk marker for impulse control behavior. An RR >200 indicates an increased risk of ICB

^a^Proposed targets based upon normal reference ranges and modified according to expert opinion

More data on the validation of both these devices in the home environment is required to further define their optimal use in OM of PD.

### Panel consensus

It is likely that treating to objectively measured targets will improve motor scores in unnecessarily undertreated, or overtreated, PwP and limit unwarranted use of medication, and therefore, improve short- and long-term clinical outcomes including QoL. Preliminary targets for each of the approved OM systems need to be established (based on the concept of aiming toward normality and expert opinion) so they can be tested in studies, and iterative improvements can be made over time.

## Why is objective measurement better than current qualitative assessment (standard of care)?

The fourth principle in Box 1 is that OM is superior to qualitative measures in determining whether a PwP is controlled or uncontrolled. Qualitative measures are currently used in the treatment of PD, but detecting variation in PD symptoms is difficult because it depends on recall, and variation may be evident to observers before they are noticed by the PwP, who may fail to report them.^48–50^ Variation in qualitative assessment according to the experience, skill, and judgment of clinicians has been reported.^8,51,52^ While movement disorder specialists (MDS) attempt to take careful histories to identify the timing and extent of clinical manifestations, there are many reasons why their best intentions are thwarted. PwP have difficulty in differentiating dyskinesia from tremor and motor fluctuations from non-motor fluctuations, patient diaries have problems with recall and “diary fatigue,”^53–55^ and failing to detect early fluctuations may constitute a lost opportunity to stabilize the treatment and to improve outcome. This emphasizes the reasons why OM are necessary in PD.^6,8^

### Panel consensus

It is likely that OM improves clinical assessment particularly for less experienced or less skilled clinicians.

## When should “objective” testing occur in PD?

In principle screening (at risk), populations using OM would identify PwP whose “uncontrolled” symptoms were unknown or undiscovered, helping in the early diagnosis of PD. Therapy for “uncontrolled” symptoms would be more effective if OM guided both the dose and choice of therapy and assessed whether control was achieved. This can be understood by comparing OM in the management of disorders such as asthma or diabetes. In these conditions OM serves three functions:

### Discovery of occult disease or symptomatology

For example, the routine measurement of blood sugar in the “normal” but at risk population for otherwise undiscovered diabetes. Even in known diabetics who are presumed to be controlled, there can be unknown, uncontrolled symptomatology such as higher average glucose levels [HbA1c], or fluctuations that result in dangerous hyper- or hypo-glycaemia. An example from PD might be to discover the presence of “wearing-off” fluctuations,^48–50^ which may be difficult to detect using current assessment strategies.^53^ The study by van der Mark et al.^51^ implies that there may be a real and sizable degree of motor and non-motor PD features in a population managed by a non-MDS. The implications for PD are that OM should be used routinely when there is a reasonable likelihood that symptoms may be “uncontrolled” even though PwP and their carers may not report problems. The need for screening for occult symptoms can be based on disease duration, treatment duration, or daily medication need.

### Objective assessment of the severity and timing of symptomatology

The assessment of the severity of known features or symptoms might include, for example, assessing the severity of fluctuations after a treatment directed at reducing their severity has been initiated. Another example might be demonstrating whether fluctuations are predictable or delayed in a manner suggesting unreliable absorption of drugs. A further example that could have significant socio-economic consequences would be to help reduce hospitalization and repeat consultations following initiation of advanced therapies via more accurate assessment of response. As discussed above, it is difficult to reliably assess symptom severity using the current standard of care.

### A means of communication

Effective and “easy-to-understand” measurement will supplement the communication between clinicians and PwP to describe a PwP’s state. Examples from other specialties are the ejection fraction in cardiology and HbA1c in diabetes. It will empower PwP by educating them about compliance with the timing of medication and individual intervention options, better recognition of their motor and non-motor symptoms, and more effective interactions with their health care providers.

An issue that is specific to PD is the need for nighttime monitoring. Polysomnography (PSG) is required for a full analysis of sleep states, but an OM can provide the percentage of time immobile, which correlates with sleep states.^56^ Thus, an OM may help identify patients that need assessment by clinical tools such as the PDSS (Parkinson’s disease sleep scale)^57^ or, if facilities permit, PSG. OM will also identify the poorly recognized early morning off periods^58^ often associated with severe non-motor symptoms and may help to optimize treatment options.

### Panel consensus

Indications for use of OM in PD should be based on the need to discover occult symptoms, guide therapy changes, and to aid communication between PwP and health professionals (Table 3). In general, OM should be used as an adjunct to any clinical assessment intended to assess a PwP need for therapy.

**Table 3:** When to use objective measurement in PD

1. Screening a (at risk) population to discover PD symptomatology that is poorly described or occult to the PwP and hence their clinician
If a PwP describes fluctuations that are not present with OM, this may indicate that they are predominantly non-motor fluctuations
PwP who have a higher risk of occult symptomatology include those who are:
at risk of dose-related (wearing-off) or unpredictable fluctuations, dyskinesia, or undertreated bradykinesia
unable to provide a clear history of symptoms
2. Objective assessment of the severity of symptomatology in PwP whose symptoms are known to be uncontrolled:
measuring the effect of a change in therapy to optimize their symptoms
assessing the severity and timing of reported symptomatology
identifying suitable candidates for advanced therapy (e.g., known fluctuations)
assessing symptomatology before or during the initiation of advanced therapy to improve titration to optimum dosages.
assessing the state of PwP who have high demands of health care resources
assessing PwP who are unable to communicate their symptom fluctuations, e.g., due to cognitive limitations
3. A means of communication. It will empower PwP by educating them about:
better recognition of their motor and non-motor symptoms
more effective interactions with their health care providers

## Practical guidance on objective measurement in routine clinical care of PD?

### What would be the ideal device for objective measurement?

The ideal measuring device in PD would detect:

- symptoms that respond to currently available therapies as well as those that result as consequences of the use of these treatments (as described in Point 2, above).
- continuously, to capture the variability of PD features over the course of the day, from day to day and with respect to the consumption of medications.
- passively during normal daily living activities, and to ensure ecological validity without requiring interrupting activities of the PwP. This also means that the instrumentation must not be onerous or intrusive to the PwP.

A key issue is whether the measurement system is recognized by regulatory bodies (for e.g., the FDA or EMA), and has CE marking to provide some assurance regarding safety, efficacy, and privacy. The latter is particularly important and brings legal obligations regarding privacy and ownership of data, which is not the case for data produced by “wearable technologies” used for personal health. In many jurisdictions, the health care provider or the prescriber must own the data and ensure its privacy until it has been anonymised. Several comprehensive reviews of devices have been recently published.^3,4,6^ It is apparent that few systems meet these ideal standards and only two have regulatory approval.^42,44^

### Panel consensus

The ideal device should have regulatory approval and continuously measure complications and therapy responsive features while PwP undertake their usual daily activities.

### Which clinicians should use objective measurement?

Before echocardiography, valvular heart disease required skilled and experienced auscultation for detection and severity assessment, and consequently most therapeutic decisions were also made by the most expert “set of ears.” Once echocardiography became ubiquitous, the detection and severity assessment was standardized and no longer depended on auscultatory competence and guidelines could be used for management and referral. Implicitly, OM will have the greatest impact on the quality of care delivered by the least experienced clinicians if they are trained appropriately.

### Panel consensus

OM should be used by all clinicians that treat PwP but the least experienced may find the most value.

### Who would interpret objective measurement in PD?

Implicit in the previous section, is that measurement will be most valuable to the least expert. However, the least experienced will also be the least capable of interpreting OM, especially if it is presented as complex data. Just as with sleep studies, EEGs, EMGs, and echocardiography, reporting is different to having targets and guidelines, and may require some sophistication and training to ensure that targets and guidelines can be sensibly and appropriately implemented. It is important that professional bodies take ownership for the training and registration of approved reporters and for this not to be left in the hands of commercial interests.

### Panel consensus

OM should be interpreted by experts to allow the non-expert to apply guidelines.

## What are the key steps necessary for the successful adoption of objective measurement?

The SP suggests guidelines for use of OM in clinical practice and proposes:

- Indications for use of the OM that will provide the most value to the health care system.
- Target scores should be defined according to the principle of aiming toward normality and using expert opinion, and trials should be undertaken to support definitions of targets and to further refine them.
- Professional bodies should consider providing guidelines as to which therapies should be used for reaching and maintaining targets.
- Responsibility for establishing training and standards of OM reporting should rest with professional bodies.

## Conclusion

The SP of movement disorder specialists was not intended to provide formal guidance on OM in PD. Rather, this guidance gives a basis for OM to be incorporated into global clinical guidelines. This is particularly important as OM devices are now available on the market for routine clinical use and guidance on the optimum use of these devices is lacking. Evidence for these devices is evolving and, in the interim, evidence-based guidance enhanced by expert opinion is needed.

### Data availability statement

No data sets were generated or analyzed in this review.
